# Phytochemical screening and evaluation of *in vitro* antioxidant and antimicrobial activities of the indigenous medicinal plant *Albizia odoratissima*

**DOI:** 10.1080/13880209.2017.1291694

**Published:** 2017-02-20

**Authors:** Venkanna Banothu, Chandrasekharnath Neelagiri, Uma Adepally, Jayalakshmi Lingam, Kesavaharshini Bommareddy

**Affiliations:** a Department of Bio-Technology, Center for Bio-Technology, Institute of Science & Technology, Jawaharlal Nehru Technological University Hyderabad, Hyderabad, Telangana, India;; b Department of Microbiology, Osmania Medical College, Hyderabad, Telangana, India

**Keywords:** Agar well diffusion, radical scavenging activity, total phenolics, total flavonoids, secondary metabolites

## Abstract

**Context:** *Albizia odoratissima* (L. f.) Benth has been used in Indian folk medicine to treat numerous inflammatory pathologies, such as leprosy, ulcers, burns and asthma.

**Objective:** To evaluate the antioxidant and antimicrobial properties of *A. odoratissima*.

**Materials and methods:** Dried leaves of *A. odoratissima* were extracted in organic solvents (hexane, chloroform, ethyl acetate, and methanol). The total phenolic content (TPC) and total flavonoid content (TFC) were determined using the Folin-Ciocalteu and aluminum chloride colorimetric methods, respectively. The antioxidant activity was examined using 2,2-diphenyl*-*1-picrylhydrazyl (DPPH), hydrogen peroxide (H_2_O_2_), 2,2′-azino-bis-(3-ethylbenzothiazoline-6-sulphonic acid) diammonium salt (ABTS), and ferric reducing antioxidant power (FRAP) assays. The antibacterial activity was examined using minimum inhibitory concentration (MIC) and the minimum bacterial concentration (MBC), determined by broth microdilution method against Gram-negative bacteria (*Klebsiella pneumoniae*, *Escherichia coli*, *Pseudomonas aeruginosa*, and *Proteus vulgaris*) and Gram-positive bacterium (*Staphylococcus aureus*).

**Results:** The TPC ranged from 4.40 ± 1.06 to 1166.66 ± 31.85 mg GAE/g of dry weight (DW), and the TFC ranged from 48.35 ± 3.62 to 109.74 ± 1.84 mg QE/g of DW. The IC_50_ values of the ethyl acetate extract for DPPH, ABTS, and H_2_O_2_ were 10.96 ± 0.40, 4.35 ± 0.07, and 163.82 ± 1.52 μg/mL, respectively. Both methanol and ethyl acetate extracts demonstrated effective antibacterial activity with MICs and MBCs values ranging 136–546 μg/mL and 273–1093 μg/mL, respectively, against the tested pathogenic species.

**Conclusions:** The leaves of *A. odoratissima* showed potent free radical scavenging property and antimicrobial activity.

## Introduction

Currently, an increasing number of studies are being conducted to explore natural compounds rich in antioxidants and antimicrobial properties because of their significance in treating various chronic disorders, such as cancer and cardiovascular disease. Approximately two-thirds of drugs approved worldwide are predicted to be plant derivatives (Patridge et al. 2016). Numerous studies support the fact that many diseases are caused by ‘oxidative stress’ resulting from a discrepancy in the neutralization and configuration of pro-oxidants. Human bodies naturally produce free radicals. Chemicals that obstruct the action of free radicals are termed as antioxidants. These overloaded free radicals contradict with biological macromolecules, such as lipids, proteins, and DNA, in healthy human cells. This results in the stimulation of carcinogenesis, cardiovascular disease, atherosclerosis, aging and inflammatory diseases (Halliwell 1996; Brahma et al. 2016).

Natural antioxidants are often added in foods to prevent the radical chain reactions of oxidation by inhibiting the initiation and propagation step leading to the termination of the reaction and a delay in the oxidation process (Hossain et al. 2011). Reactive oxygen species, such as the superoxide anion (O_2_^−^), hydroxyl radicals (OH^−^), and nitric oxide (NO), inactivate enzymes and damage vital cellular components, causing injury. Antioxidants may provide resistance against oxidative stress by scavenging free radicals. Therefore, compounds with antioxidative properties may be useful in the treatment of various disorders (Parim et al. 2015).

Medicinal plants have been widely utilized as substitute agents for curing diverse infections and diseases for decades. The materialization of drug resistance in animal and human pathogenic bacteria, as well as the unwanted side effects of some antibiotics, has attracted massive attention toward the exploration of novel antimicrobial alternatives of the plant origin. The most significant benefits claimed for remedial use of medicinal plants in diverse ailments are their efficacy, cost-effective nature and easy accessibility (Khan et al. 2009; Mallikarjun et al. 2016).

*Albizia odoratissima* (L. f.) Benth (Leguminosae), commonly known as ‘Black Siris’ and ‘Ceylon rosewood,’ is a fast-growing deciduous tree. *Albizia* has been used in Indian folk medicine to treat numerous inflammatory pathologies, such as arthritis, dysentery, sepsis, burns, asthma, allergic rhinitis, and helminth infections (Higuchi et al. 1992; Dinesh et al. 2011). Thus, the present study examined the antioxidant and antimicrobial activities of different solvent extracts prepared from the leaves of *A. odoratissima* on the basis of their phytochemical significance.

## Materials and methods

### Chemicals and reagents

2,2′-Azino-bis-(3-ethylbenzothiazoline-6-sulphonic acid) diammonium salt (ABTS), potassium persulfate (K_2_S_2_O_8_), 2, 2-diphenyl*-*1-picrylhydrazyl (DPPH), hydrogen peroxide (H_2_O_2_), 2, 4, 6-tripyridyl-s-triazine (TPTZ), gallic acid, quercetin, butylated hydroxyl toluene (BHT), ascorbic acid, Mueller–Hinton agar, and broth were obtained from Sigma-Aldrich Chemicals (St. Louis, MO). All other chemicals were of the analytical grade and were purchased from Merck Limited (Mumbai, India).

### Preparation of extracts

The leaves of *A. odoratissima* were collected from the Eastern Ghats, Visakhapatnam, Andhra Pradesh, India, in March 2013. The identity of these leaves was authenticated at Regional Agriculture Research Centre, Guntur, Andhra Pradesh, India, and a specimen has been preserved in the departmental herbarium (Voucher number: AB#2054). Leaves were shade dried and ground into a coarse powder. The powder (200 g) was soaked sequentially in hexane, chloroform, ethyl acetate, and methanol each for 6–10 days at room temperature in a 10 L aspirator jar to collect extracts. These extracts were concentrated using a rotavaporator for further analyses. All the extracts were preserved in a refrigerator at 4 °C.

### Determination of the total phenolic content

The TPC was determined using the Folin–Ciocalteu (FC) method (Djeridane et al. 2006) with a slight modification. The calibration curve was constructed using gallic acid (20–500 μg/mL) as a standard. Briefly, 1 mL of the crude extract was diluted up to 3 mL with distilled water and mixed thoroughly with 1 mL of the FC reagent (previously diluted 6-fold with distilled water), followed by the addition of 2 mL of 20% (w/v) sodium carbonate. The mixture was allowed to stand for 30 min in dark, and absorbance was measured at 765 nm by using an UV–visible spectrophotometer (Shimadzu, Japan). The TPC was evaluated from a calibration curve, and the results are expressed as milligrams (mg) of gallic acid equivalents per gram (g) of a leaf (dry weight).

### Determination of the total flavonoid content

The TFC of the crude leaf extracts was determined using the aluminum chloride colorimetric method (Zhishen et al. 1999) with a slight modification. Quercetin (20–100 μg/mL) was used as a standard to plot the calibration curve. Briefly, 1 mL of the crude extract was mixed with 2.8 mL of double distilled water and then with 0.1 mL of 1 mg/mL potassium acetate solution. To this solution, 0.1 mL of 10% aluminum chloride was added, the mixture was allowed to stand for 30 min, and absorbance was measured at 415 nm by using a UV–visible spectrophotometer (Shimadzu). The TFC was evaluated from a calibration curve, and the results are expressed as milligrams (mg) of quercetin equivalents per gram (g) of a leaf (dry weight).

### DPPH radical scavenging activity assay

The DPPH assay is a simple, rapid, economical, and widely used method to evaluate antioxidant activity. Although the DPPH assay involves the transfer of hydrogen atoms, the underlying chemical reaction is considered to be an electron transfer (ET) reaction. This is because the transfer of hydrogen from an antioxidant to DPPH is a very slow process and is considered as a marginal reaction path, whereas ET from a deprotonated antioxidant to DPPH is a faster and rate-determining step.

The free radical scavenging activity of all the extracts was evaluated using a method described by Gyamfi et al. (1999). Five concentrations (1 mL) of test samples were mixed with 1 mL of 0.2 mM DPPH in ethanol solution. After shaking vigorously, the mixture was incubated in dark at room temperature for 30 min, and the absorbance was measured at 517 nm. Ascorbic acid and BHT were used as standards, whereas ethanol was used as a control for calculation. The radical scavenging activity of all the extracts was calculated as the percentage inhibition of absorbance by using the following formula and IC_50_ values were determined.

$$\%\;\mathrm{radical}\;\mathrm{scavenging}\;\mathrm{activity}\;=\;\left[\left({\mathrm{OD}}_{\mathrm{control}}-{\mathrm{OD}}_{\mathrm{sample}}\right)/{\mathrm{OD}}_{\mathrm{control}}\right]\times 100,$$

where optical density (OD) is the absorbance of the sample.

### Free radical scavenging ability by the use of a stable ABTS radical cation assay

The ABTS assay can be used to determine the antioxidant capacity of both hydrophilic and lipophilic samples because ABTS^*+^ is soluble in water and organic solvents. In the presence of hydrogen-donating antioxidants, the blue/green ABTS^*+^ is reduced to colorless ABTS at 734 nm. The antioxidant activity is proportional to the decrease in the absorbance (Re et al. 1999).

The total antioxidant activity of the test sample was measured according to the method of Hsu et al. (2011), with a slight modification. ABTS^*+ ^solution was prepared by mixing 7 mM of ABTS and 2.45 mM of K_2_S_2_O_8_ in water, which was incubated for 12–16 h in dark at room temperature. Before use, the ABTS solution was diluted with a solution of water and ethanol to get an absorbance of 0.7 ± 0.02 at 734 nm by using a UV-visible spectrophotometer (Shimadzu). Briefly, 2 mL of the ABTS^*+ ^solution was added to 100 μL of test samples at different concentrations. The samples were mixed thoroughly, the reaction mixtures were incubated at room temperature for 10 min, and the absorbance was recorded immediately at 734 nm. The percentage inhibition of absorbance was calculated and plotted as a function of the concentration of the standard and sample. IC_50_ values were determined. The percentage inhibition of absorbance was calculated using the following formula:

$$\%\;\mathrm{radical}\;\mathrm{scavenging}\;\mathrm{activity}\;=\;\left[\left({\mathrm{OD}}_{\mathrm{control}}-{\mathrm{OD}}_{\mathrm{sample}}\right)/{\mathrm{OD}}_{\mathrm{control}}\right]\times 100,$$

where OD is the absorbance of the samples.

### H_2_O_2_ scavenging capacity assay

H_2_O_2_ is a weak oxidizing agent and can directly inactivate a few enzymes, usually through the oxidation of essential thiol (-SH) groups. H_2_O_2_ can rapidly cross cell membranes and enter into a cell. H_2_O_2_ probably reacts with Fe^2+ ^and possibly with Cu^2+ ^ions to form a hydroxyl radical, which may be the origin of its many toxic effects (Miller et al. 1993). Therefore, it is biologically advantageous for cells to control the amount of H_2_O_2_ that is allowed to accumulate.

The ability of the extracts to scavenge H_2_O_2_ was determined using the method described by Owaisi et al. (2014), with a slight modification. A solution of H_2_O_2_ (40 mM) was prepared in phosphate buffer (0.05 mM, pH 7.4). Plant extracts (1 mL) at different concentrations were mixed with H_2_O_2_ solution (0.6 mL, 40 mM). The absorbance of H_2_O_2_ was determined at 230 nm by using a UV–visible spectrophotometer (Shimadzu, Japan) after 10 min against a blank solution containing phosphate buffer without H_2_O_2_. The percentage inhibition of H_2_O_2_ scavenging by the extracts and a standard compound was calculated using the following equation, and IC_50_ values were determined.

$$\%\;\mathrm{radical}\;\mathrm{scavenging}\;\mathrm{activity}\;=\;\left[\left({\mathrm{OD}}_{\mathrm{control}}-{\mathrm{OD}}_{\mathrm{sample}}\right)/{\mathrm{OD}}_{\mathrm{control}}\right]\times 100,$$

where OD is the absorbance of samples.

### Ferric reducing antioxidant power activity assay

The ferric reducing antioxidant power (FRAP) assay is based on the ability of antioxidants to reduce (through an ET mechanism) Fe^3+ ^into Fe^2+ ^ions in the presence of TPTZ, forming an intense blue Fe^2+^–TPTZ complex with the absorption maximum at 593 nm. The reaction is pH dependent (optimum pH, 3.6).

The FRAP assay was performed using the method described by Nair et al. (2007). The FRAP solution was prepared by mixing 100 mL of acetate buffer at 30 mM, 10 mL of a 10 mM TPTZ [2,4,6-tripyridyl-s-triazine] in 40 mM HCL, and 10 mL of FeCl_3_.6H_2_O at 20 mM. Then, 100 μL of the crude extract (100–500 μg/mL) was mixed with 3 mL of the FRAP solution, and the reaction mixture was incubated at 37 °C for 30 min. The increase in the absorbance at 593 nm was measured. Fresh working solutions of FeSO_4_ were used for calibration. The antioxidant capacity was calculated on the basis of the ability of the sample to reduce ferric ions from the linear calibration curve and expressed as millimoles (mmol) of FeSO_4_ equivalents per gram dry weight of the sample.

### Antibacterial activity assay

#### Microorganisms and media

Bacterial strains were procured from the Department of Microbiology, Osmania General Hospital, Hyderabad, India. The cultures included in this study were the Gram-negative bacteria *Klebsiella pneumoniae* (U98), *Escherichia coli* (E72), *Pseudomonas aeruginosa* (3023), and *Proteus vulgaris* (2266) and the Gram-positive bacteria *Staphylococcus aureus* (E40), which were maintained in Mueller-Hinton agar slants at 4 °C.

### Minimum inhibitory concentration and minimum bactericidal concentration

The minimum inhibitory concentration (MIC) was determined using the microbroth dilution method performed in 96-well plates according to the standard protocol (Shariff et al. 2006; NCCLS 2008) with some modification. A 2-fold serial dilution of the crude extracts, with an appropriate antibiotic, was prepared. Ceftriaxone (1 mg/mL) was used as a positive control. Initially, 100 μL of the MH broth was added to each well. Then, 100 μL of the crude extract or an antibiotic was taken from the stock solution and dissolved in the first well. Serial dilution was performed to obtain different concentrations. The concentrations of the stock solution of methanol, ethyl acetate, chloroform, and hexane extracts were 70, 70, 110, and 120 mg/mL, respectively. Furthermore, 24 h culture turbidity was adjusted to match 0.5 McFarland standards, which correspond to 1 × 10^8^ CFU/mL. The standardized suspension (100 μL) of bacteria was added to all the wells except the antibiotic control well, and 96-well plates were incubated at 37 °C for 24 h. After 24 h of incubation, 40 μL of 3-(4,5-dimethlthiazol-2-yl)-2,5-diphenyltrazolium bromide (MTT) reagent (0.5 mg/mL in 1 × PBS) was added to all the wells. MIC was taken as the lowest concentration, which did not show any growth that is visually noted from the blue color developed by MTT. Subcultures were prepared from clear wells, and the lowest concentration that yielded no growth after subculturing was taken as the MBC.

### Statistical analysis

All assays of the TPC, flavonoid compounds, and antioxidant activity (using different assays) were performed in triplicates. Values for each sample are expressed as the mean ± standard deviation and were subjected to analysis of variance. Statistical analysis was conducted using the Graph Pad Prism Software, Version 7.01 (GPPS Inc., La Jolla, CA). Correlations between the means were assessed using Dunnett’s multiple comparison test. For antioxidant assays, 1/IC_50_ value was used to determine the correlation, and *p* < 0.05 was considered statistically significant.

## Results

### Determination of the TPC

**Figure 1.**
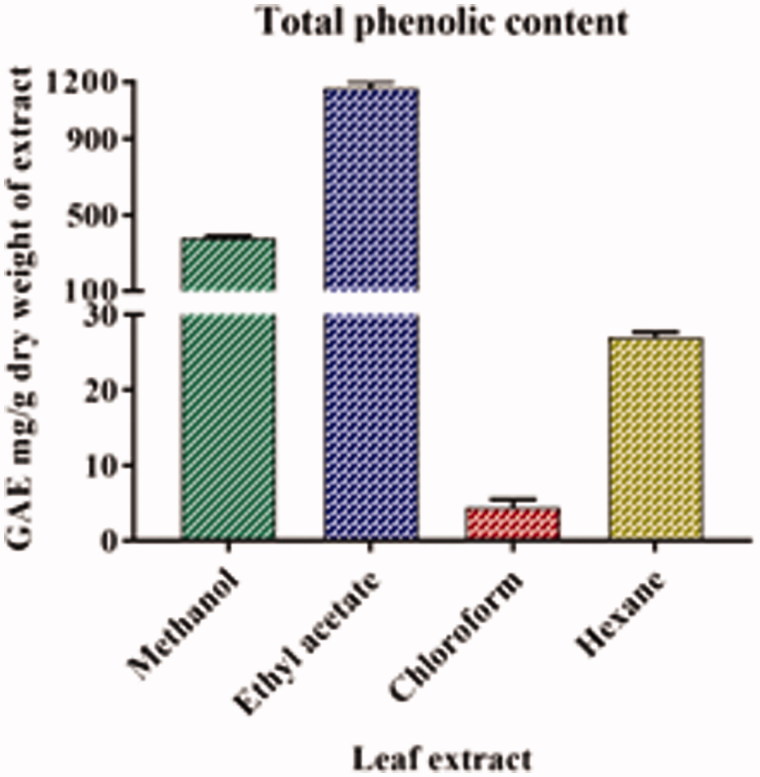
Total phenolic content in various leaf extracts of *A. odoratissima*. The *y*-axis was cut up from 30 to 100 for better understanding.

The TPC of all the four extracts of *A. odoratissima* are listed in Table 1. The ethyl acetate extract of the leaf of *A. odoratissima* had the highest amount of phenolic compounds, followed by methanol, hexane, and chloroform extracts. The results of the analytical method were validated by a linear correlation between the concentration and absorbance, and an *R* ^2^ value of 0.996 was obtained (Figure 1).

**Table 1.** Total phenolic content and total flavonoid content of various leaf extracts of *Albizia odoratissima*.

		Equivalents per g dry weight of extract (mg/g)	
S. No.	Name of extracts	Total phenolic content (Gallic acid)	Total flavonoid content (Quercetin)
1	Methanol	378.00 ± 11.50	48.35 ± 3.62
2	Ethyl acetate	1166.66 ± 31.85	87.31 ± 3.70
3	Chloroform	4.40 ± 1.06	74.93 ± 4.98
4	Hexane	27.01 ± 0.67	109.74 ± 1.84
5	*p* value	*p* < 0.05	*p* < 0.05

The data represent the mean ± SD of three determinants and level of significance (*p*).

### Determination of the TFC

**Figure 2.**
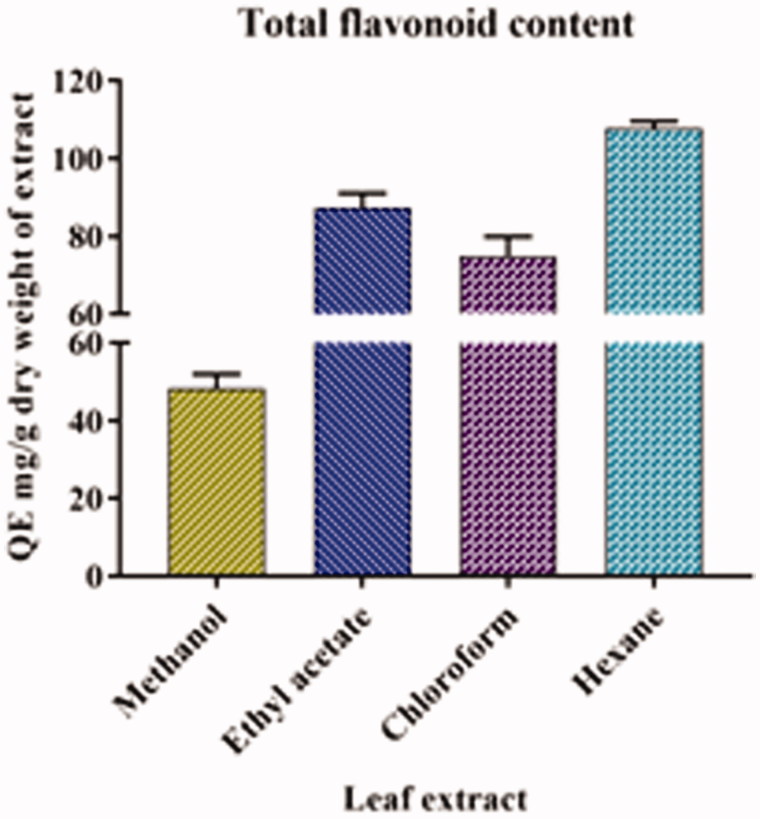
Total flavonoid content in various leaf extracts of *A. odoratissima*.

The TFC of all the four extracts of *A. odoratissima* are listed in Table 1. The hexane extract had the highest amount of flavonoid content, followed by ethyl acetate, chloroform, and methanol extracts. The results of the analytical method were validated by a linear correlation comparison, and an *R* ^2^ value of 0.995 was obtained (Figure 2).

### Free radical scavenging effect on the DPPH assay

**Figure 3.**
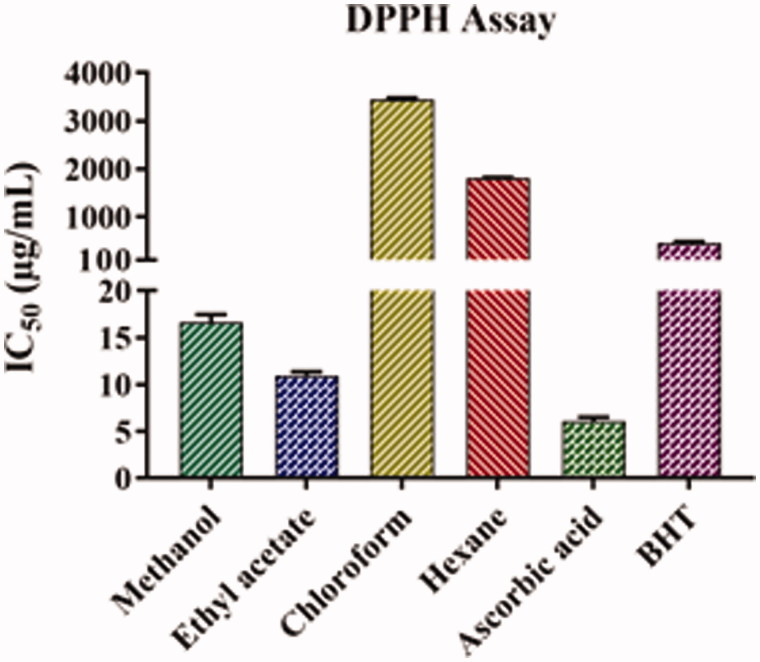
DPPH antioxidant activity of leaf extracts of *Albizia odoratissima*. The *y*-axis was cut up from 20 to 100 for better understanding.

The results of the free radical scavenging activity of the leaf extracts of *A. odoratissima* are shown in Figure 3 and Table 2. A lower IC_50_ value indicates higher antioxidant activity. Among all the extracts, ethyl acetate had the lowest IC_50_ value (10.96 ± 0.40 μg/mL), followed by methanol (16.62 ± 0.85 μg/mL), hexane (1810.44 ± 13.50 μg/mL), and chloroform (3439.66 ± 33.56 μg/mL). Furthermore, the IC_50_ values of ethyl acetate and methanol extracts were comparable to those of ascorbic acid (6.11 ± 0.44 μg/mL) but lower than that of BHT (458.10 ± 33.09 μg/mL), suggesting that the antioxidant activity of these extracts is higher than that of BHT.

**Table 2.** Free radical scavenging activity of different leaf extracts of *Albizia odoratissima*.

			Percentage (%) of inhibitory effect (IC_50_; μg/mL)		
S. No.	Sample	Extractions	DPPH	ABTS	H_2_O_2_
1	Ascorbic acid	–	6.11 ± 0.44	3.65 ± 0.26	860.23 ± 26.83
2	BHT	–	458.10 ± 33.09*	6.57 ± 0.57	912.46 ± 161.16
3	Leaf	Methanol	16.62 ± 0.85	8.59 ± 0.39	200.29 ± 0.70*
4	Leaf	Ethyl acetate	10.96 ± 0.40	4.35 ± 0.07	163.82 ± 1.52*
5	Leaf	Chloroform	3439.66 ± 33.56*	58.81 ± 3.69	6912.30 ± 156.88*
6	Leaf	Hexane	1810.44 ± 13.50*	52.08 ± 1.26	1278.64 ± 131.83*

The data represent the mean ± SD of three determinants and level of significance.

* *p* < 0.05, ascorbic acid is used as control for statistical significance analysis.

### Free radical scavenging ability by the use of a stable ABTS radical cation assay

**Figure 4.**
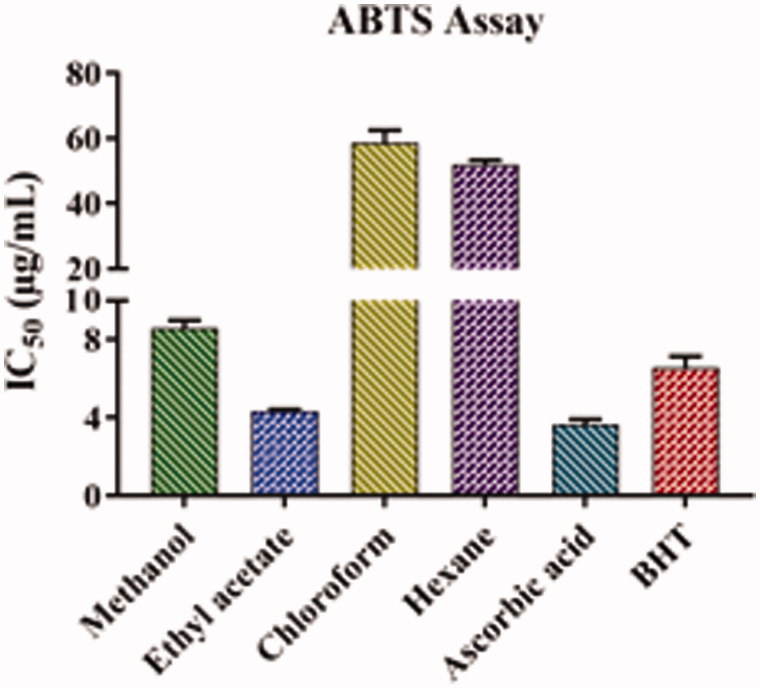
ABTS antioxidant activity of leaf extracts of *Albizia odoratissima*. The *y*-axis was cut up from 10 to 20 for better understanding.

The results of the free radical scavenging activity of the leaf extracts of *A. odoratissima* are presented in Figure 4 and Table 2. The decreasing order of the ABTS scavenging activity of different extracts was as follows: ethyl acetate (4.35 ± 0.07 μg/mL), methanol (8.59 ± 0.39 μg/mL), hexane (52.08 ± 1.26 μg/mL), and chloroform (58.81 ± 3.69 μg/mL). The IC_50_ value of the ethyl acetate extract was almost equal to that of ascorbic acid (3.65 ± 0.26 μg/mL) and lower than that of BHT (6.57 ± 0.57 μg/mL), indicating that the ethyl acetate extract has strong antioxidant activity. In addition, the antioxidant activity of the methanolic extract was slightly lower than that of BHT and ascorbic acid.

### H_2_O_2_ scavenging capacity assay

**Figure 5.**
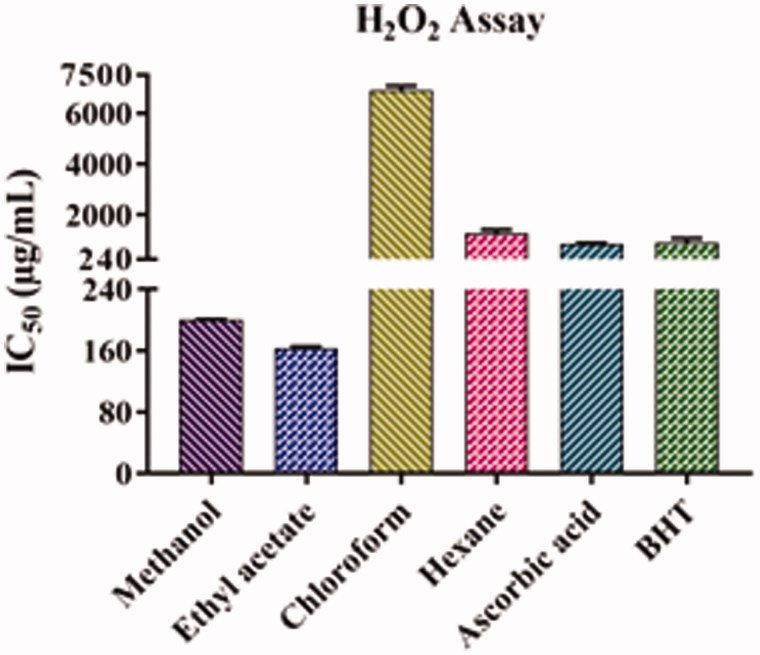
Hydrogen peroxide activity of leaf extracts of *Albizia odoratissima*.

The results of the free radical scavenging activity of the leaf extracts of *A. odoratissima* are shown in Figure 5 and Table 2. The ethyl acetate extract (163.82 ± 1.52 μg/mL) showed the highest H_2_O_2_ scavenging activity, followed by methanol (200.29 ± 0.70 μg/mL), ascorbic acid (860.23 ± 26.83 μg/mL), BHT (912.46 ± 161.16 μg/mL), hexane (1278.64 ± 131.83 μg/mL), and chloroform (6912.30 ± 156.88 μg/mL) extracts. Furthermore, the antioxidant activity of ethyl acetate and methanol extracts was higher than that of ascorbic acid and BHT, which was indicated by their lowest IC_50_ values.

**Figure 6.**
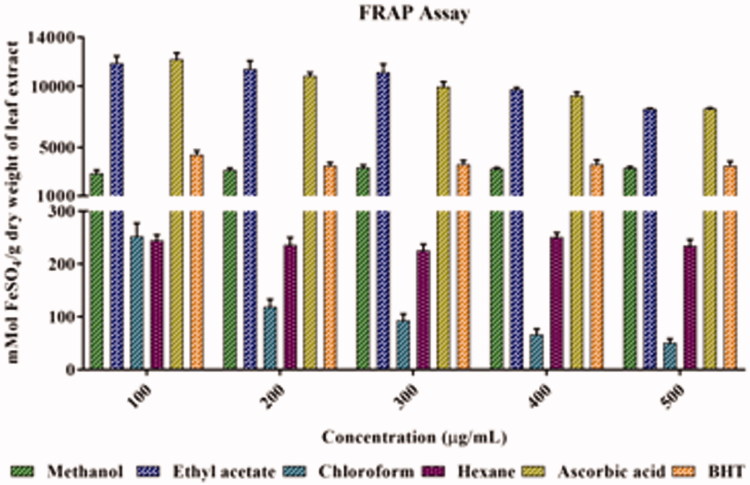
Ferric reducing antioxidant power activity of leaf extracts of *Albizia odoratissima*. The *y*-axis was cut up from 300 to 1000 for better understanding.

### FRAP activity

Regarding the antioxidant capacity of the leaf extracts of *A. odoratissima*, the ethyl acetate extract showed the maximum reducing power, followed by methanol, hexane, and chloroform extracts. In addition, the reducing power of ethyl acetate extract was comparable to that of ascorbic acid. The methanol extract also demonstrated reducing power, which was comparable to that of BHT. The results of the analytical method were validated by a linear correlation comparison of ferrous sulfate, and an *R* ^2^ value of 0.996 (Figure 6) was obtained.

### Correlation between different assays

A significant positive correlation was observed between the TPC and antioxidant activity determined by DPPH (*r* = 0.939, *p* > 0.05), H_2_O_2_ (*r* = 0.884, *p* > 0.05), ABTS (*r* = 0.988, *p* < 0.05), and FRAP (*r* = 0.996, *p* < 0.05) assays. In addition, a strong positive correlation was observed ranging from 0.921 to 0.997 among antioxidant activities determined using different assays (Table 3). Furthermore, a negative correlation was observed between the TFC and other antioxidant assays ranging from −0.401 to −0.137. The correlation between the TFC and other antioxidant assays was not significant.

**Table 3.** Correlation matrix of total phenolic content, total flavonoid content and antioxidant activity measured by all the four *in vitro* assays.

	TPC	TFC	DPPH	H_2_O_2_	ABTS	FRAP
TPC	1					
TFC	−0.067	1				
DPPH	0.939	−0.355	1			
H_2_O_2_	0.884	−0.401	0.988*	1		
ABTS	0.988*	−0.200	0.981*	0.945	1	
FRAP	0.996*	−0.137	0.965*	0.921	0.997*	1

Level of significance: *p < 0.05.

HE: Hexane; CH: Chloroform; EA: Ethyl acetate; ME: Methanol and IC: Isolated colony; NI: No inhibition. Inhibitory zones in mm, including diameter of the well (8 mm) showing the best value among the three repeats.

### Antibacterial activity

In the microbroth dilution assay, ethyl acetate and methanol extracts showed satisfactory antimicrobial effects against the test organisms. The MICs and MBCs are listed in Table 4. The MICs of the methanol and ethyl acetate extracts ranged from 136 to 546 μg/mL, and the MBCs ranged from 273 to 1093 μg/mL against all the tested microorganisms.

**Table 4.** MIC and MBC of different leaf extracts of *Albizia odoratissima* against the tested pathogenic species.

	Different solvent extracts (μg/mL)									
	ME		EA		CH		HE		Ceftriaxone	
Strains	MIC	MBC	MIC	MBC	MIC	MBC	MIC	MBC	MIC	MBC
*S. aureus*	136	273	273	546	3437	6875	15,000	30,000	62	125
*E. coli*	273	546	136	273	859	1718	15,000	30,000	31	62
*K. pneumoniae*	273	546	273	546	6875	13,750	7500	15,000	62	125
*P. vulgaris*	546	1093	136	273	6875	13,750	15,000	30,000	62	125
*P. aeruginosa*	546	1093	546	1093	1718	3437	15,000	30,000	62	125

HE: Hexane; CH: Chloroform; EA: Ethyl acetate; ME: Methanol and MIC: Minimum inhibitory concentration; MBC: Minimum bactericidal concentration. The table represents the best MIC and MBC value among the three repeats.

The leaf extracts exhibited antibacterial activity against all the pathogenic species. Methanol and ethyl acetate extracts exhibited almost similar antimicrobial activity against all the pathogens. However, hexane and chloroform extracts showed very less or no antimicrobial effect.

## Discussion

In recent days, people attempted to reduce a hazard or handle a specific health situation through enhanced food diet. Plants and fruits have evolved diverse phytochemicals, which have a high amount of antioxidant potential. Natural antioxidants are considered to be multifunctional and of high interest as alternatives to synthetic antioxidants to reduce oxidation in complex food systems (Wang et al. 2009).

Phytochemical analyses performed on plant extracts have shown the presence of constituents, which have been identified to demonstrate therapeutic and physiological activities (Sofowora 1993). Numerous studies have reported the antioxidant properties of phenolic compounds present in diverse parts of various medicinal plants (Krings & Berger 2001). The imperative function of antioxidants is their interface with oxidative free radicals. Phenolic compounds, such as terpenoids and flavonoids, are the principal antioxidants that exert a scavenging effect on free radicals and reactive oxygen species (Hossain et al. 2011). The antioxidative properties of flavonoids are due to numerous dissimilar mechanisms including the chelation of metal ions, such as iron and copper; the scavenging of free radicals; and the inhibition of enzymes responsible for free radical generation (Benavente et al. 1997).

The results of the current study demonstrated that the leaf extracts of *A. odoratissima* possess strong free radical scavenging activity. The four different extracts of the leaves of *A. odoratissima* contain a wide variety and quantity of phenolics and adequate quantities of flavonoid components (Hossain et al. 2011).

In the present study, the four organic extracts of *A. odoratissima* could decolourize the free radicals. DPPH, ABTS, and H_2_O_2_ scavenging assays were used to determine the antioxidant activity (Mathew & Abraham 2006). The antioxidant activities of the extracts could be due to increased levels of polyphenols. The results indicate that compared with methanol, hexane, and chloroform extracts, the ethyl acetate extract of the leaves of *A. odoratissima* demonstrated superior activity. The antioxidant capacity of the four extracts of *A. odoratissima* was also determined using the FRAP assay. The results of the FRAP assay indicated that the ethyl acetate extract of the leaf exhibited the maximum reducing power. Because *A. odoratissima* possesses antioxidant activity, it can be vital to human health (Halliwell 1996).

We evaluated the antimicrobial activity of *A. odoratissima* plant extracts and determined the MIC and MBC. The extracts of *A. odoratissima* were found to possess strong antimicrobial activity (Table 4). The antimicrobial effectiveness of plants is supposed to be due to the presence of phenolic compounds, tannins, flavonoids, and essential oils (Aboaba & Efuwape 2001). Moreover, even crude extracts of these plants demonstrated strong activity against multidrug resistant strains. Our findings revealed that the MIC values of the extracts were lower than their MBC values, indicating a strong antimicrobial activity.

The results of this study revealed that methanol and ethyl acetate extracts inhibited the growth of *K. pneumoniae*, *E. coli, P. aeruginosa*, *P. vulgaris* and *S. aureus*. The MIC of the methanol and ethyl acetate extracts ranged from 136 to 546 μg/mL, whereas that of chloroform and hexane extracts ranged from 859 to 15000 μg/mL. However, the chloroform and hexane extracts of *A. odoratissima* could not inhibit the growth of pathogens. The dissimilarity in the sensitivity of the tested microorganisms can be attributed to their inherent properties that are connected to the permeability of their cell exterior to the extracts or the solubility of the active compounds in the given solvents.

## Conclusions

The leaves of *A. odoratissima*, which contain a high amount of flavonoids and phenolic compounds, exhibit high antioxidant and free radical scavenging activities. The *in vitro* assays demonstrated that this plant extract is a noteworthy resource of an innate antioxidant, which may be help in preventing the development of diverse oxidative stresses. The results of our study revealed superior antioxidant and antimicrobial activities when ethyl acetate and methanol extracts of *A. odoratissima* were utilized. These results support the notion that a diet rich in herbs and plants can possibly reduce oxidation and microbial growth and act as a defense against associated disorders. Although our findings indicate that *A. odoratissima* possesses antimicrobial and antioxidant properties, further research on the isolation and formulation of active ingredients from the leaves should be conducted for its therapeutic applications.

## References

[CIT0001] Aboaba O, Efuwape BM. 2001 Antibacterial properties of some Nigerian species. Bio Res Comm. 13:183–188.

[CIT0002] Benavente GO, Castillo J, Marin FR. 1997 Uses and properties of *Citrus* flavonoids. J Agric Food Chem. 45:4505–4515.

[CIT0003] Brahma NP, Uddandarao VV, Ravindar Naik R, Suresh P, Meriga B, Begum MS, Pandiyan R, Saravanan G 2016 Ameliorative potential of gingerol: Promising modulation of inflammatory factors and lipid marker enzymes expression in HFD induced obesity in rats. Mol Cell Endocrinol. 419:139–147. 26493465 10.1016/j.mce.2015.10.007

[CIT0004] Dinesh K, Sonia K, Sunil K, Jyoti G, Pranay J, Ram KP 2011 Screening of methanolic bark extract of *Albizia odoratissima* for antimicrobial activity. Phcog Commn. 1:47–49.

[CIT0005] Djeridane A, Youffi M, Nadjemi B. 2006 Antioxidant activity of some Algerian medicinal plants extracts containing phenolic compounds. Food Chem. 97:654–660.

[CIT0006] Gyamfi MA, Yonamine M, Aniya Y. 1999 Free radical scavenging activity of medicinal herb of Ghana: *Thonningia sanguinea* on experimentally induced liver injuries. Gen Pharmacol. 32:661–667. 10401991 10.1016/s0306-3623(98)00238-9

[CIT0007] Halliwell B. 1996 Antioxidants in human health and disease. Annu Rev Nutr. 16:35–50. 10.1146/annurev.nu.16.070196.000341 8839918

[CIT0008] Higuchi H, Kinjo J, Nohara T. 1992 An arrhythmic-inducing glycoside from *Albizia julibrissin* Durazz. IV. Chem Pharm Bull. 40:829–831. 1611699 10.1248/cpb.40.829

[CIT0010] Hossain MA, Shah MD, Gnanaraj C, Iqbal M. 2011 *In vitro* total phenolics, flavonoids contents and antioxidant activity of essential oil, various organic extracts from the leaves of tropical medicinal plant *Tetrastigma* from Sabah. Asai Paci J Tropi Medic. 4:717–721. 10.1016/S1995-7645(11)60180-6 21967695

[CIT0011] Hsu CF, Peng H, Basle C, Sejdic JT, Kilmartin PA 2011 ABTS^*+^ scavenging activity of polypyrrole, polyaniline and poly (3,4-ethylenedioxythiophene). Polym Int. 60:69–77.

[CIT0012] Krings U, Berger RG. 2001 Antioxidant activity of roasted foods. Food Chem. 72:223–229.

[CIT0013] Mallikarjun S, Ashwini R, Rajesh G, Ramya S, Mithun P 2016 Antimicrobial efficacy of Tulsi leaf (*Ocimum sanctum*) extract on periodontal pathogens: An *in vitro* study. J Indian Soc Periodontol. 20:145–150. 27143825 10.4103/0972-124X.175177 PMC4847459

[CIT0014] Mathew S, Abraham TE. 2006 *In vitro* antioxidant activity and scavenging effects of *Cinnamomum verum* leaf extract assayed by different methodologies. Food Chem Toxicol. 44:198–206. 16087283 10.1016/j.fct.2005.06.013

[CIT0015] Miller WR, Benefield RG, Tonigan JS. 1993 Enhancing motivation for change in problem drinking: a controlled comparison of two therapist styles. J Consult Clin Psychol. 61:455–461. 8326047 10.1037//0022-006x.61.3.455

[CIT0016] Owaisi MA, Hadiwi NA, Khan SA. 2014 GC-MS analysis, determination of total phenolics, flavonoid content and free radical scavenging activities of various crude extracts of *Moringa peregrine* (Forssk.) Fiori leaves. Asai Pacific J Tropical Biomedici. 4:964–970.

[CIT0017] Nair VDP, Dairam A, Agbonon A, Arnason JT, Foster BC, Kanfer I. 2007 Investigation of the antioxidant activity of African potato (*Hypoxis hemerocallidea*). J Agric Food Chem. 55:1707–1711. 17295502 10.1021/jf0619838

[CIT0018] NCCLS 2008 Performance standards for antimicrobial susceptibility testing. Ninth informational supplement. NCCLS document M100-S9. Wayne (PA): National Committee for Clinical Laboratory Standard.

[CIT0020] Patridge E, Gareiss P, Kinch MS, Hoyer D 2016 An analysis of FDA-approved drugs: natural products and their derivatives. Drug Discov Today. 21:204–207. 25617672 10.1016/j.drudis.2015.01.009

[CIT0021] Parim B, Harishankar N, Balaji M, Pothana S, Sajjalaquddam RR 2015 Effects of *Piper nigrum* extracts: Restorative perspectives of high fat diet induced changes on lipid profile, body composition, and hormones in sprague-dawley rats. Pharm Biol. 53:1318–1328. 25856709 10.3109/13880209.2014.980585

[CIT0022] Re R, Pellegrini N, Proteggente A, Pannala A, Yang M, Rice-Evans C 1999 Antioxidant activity is applying an improved ABTS radical cation decolorization assay. Free Radic Biol Med. 26:1231–1237. 10381194 10.1016/s0891-5849(98)00315-3

[CIT0023] Khan R, Islam B, Akram M, Shakil S, Ahmad A, Ali SM, Siddiqui M, Khan AU 2009 Antimicrobial activity of five herbal extracts against multi drug resistant (MDR) strains of bacteria and fungus of clinical origin. Molecules. 14:586–597. 19214149 10.3390/molecules14020586 PMC6253777

[CIT0024] Shariff N, Sudarshana MS, Umesha S, Hariprasad P 2006 Antimicrobial activity of *Rauvolfia tetraphylla* and *Physalis minima* leaf and callus extracts. Afric J Biotechn. 5:946–950.

[CIT0026] Sofowora A. 1993 Medicinal plants and traditional medicine in Africa. New York: John Wiley and Sons Ltd; p. 191–289.

[CIT0027] Wang T, Jónsdóttir R, Ólafsdóttir G. 2009 Total phenolic compounds, radical scavenging and metal chelation of extracts from Icelandic seaweeds. Food Chem. 116:240–248.

[CIT0028] Zhishen J, Mengcheng T, Wu J. 1999 The determination of flavonoid content in mulberry and their scavenging effects on superoxide radicals. Food Chem. 64:555–559.

